# Social determinants of influenza hospitalization in the United States

**DOI:** 10.1111/irv.12483

**Published:** 2017-10-06

**Authors:** Rameela Chandrasekhar, Chantel Sloan, Edward Mitchel, Danielle Ndi, Nisha Alden, Ann Thomas, Nancy M. Bennett, Pam D. Kirley, Mary Hill, Evan J. Anderson, Ruth Lynfield, Kimberly Yousey‐Hindes, Marisa Bargsten, Shelley M. Zansky, Krista Lung, Monica Schroeder, Maya Monroe, Seth Eckel, Tiffanie M. Markus, Charisse N. Cummings, Shikha Garg, William Schaffner, Mary Lou Lindegren

**Affiliations:** ^1^ Vanderbilt University School of Medicine Nashville TN USA; ^2^ Brigham Young University Provo UT USA; ^3^ Colorado Department of Public Health and Environment Denver CO USA; ^4^ Oregon Department of Public Health Portland OR USA; ^5^ University of Rochester School of Medicine and Dentistry Rochester NY USA; ^6^ California Emerging Infections Program Oakland CA USA; ^7^ Salt Lake County Health Department Salt Lake City CO USA; ^8^ Georgia Emerging Infections Program Atlanta VAMC Emory University Atlanta GA USA; ^9^ Minnesota Department of Health St. Paul MN USA; ^10^ Connecticut Emerging Infections Program Yale School of Public Health New Haven CT USA; ^11^ New Mexico Department of Health Santa Fe NM USA; ^12^ New York State Department of Health Albany NY USA; ^13^ Ohio Department of Health Columbus OH USA; ^14^ Council of State and Territorial Epidemiologists Atlanta GA USA; ^15^ Maryland Department of Health and Mental Hygiene Baltimore MD USA; ^16^ Michigan Department of Health and Human Services Lansing MI USA; ^17^ Influenza Division CDC Atlanta GA USA

**Keywords:** census tract‐based determinants, disparities, geocoding, influenza hospitalization, multilevel modeling, socioeconomic determinants

## Abstract

**Background:**

Influenza hospitalizations result in substantial morbidity and mortality each year. Little is known about the association between influenza hospitalization and census tract‐based socioeconomic determinants beyond the effect of individual factors.

**Objective:**

To evaluate whether census tract‐based determinants such as poverty and household crowding would contribute significantly to the risk of influenza hospitalization above and beyond individual‐level determinants.

**Methods:**

We analyzed 33 515 laboratory‐confirmed influenza‐associated hospitalizations that occurred during the 2009‐2010 through 2013‐2014 influenza seasons using a population‐based surveillance system at 14 sites across the United States.

**Results:**

Using a multilevel regression model, we found that individual factors were associated with influenza hospitalization with the highest adjusted odds ratio (AOR) of 9.20 (95% CI 8.72‐9.70) for those ≥65 vs 5‐17 years old. African Americans had an AOR of 1.67 (95% CI 1.60‐1.73) compared to Whites, and Hispanics had an AOR of 1.21 (95% CI 1.16‐1.26) compared to non‐Hispanics. Among census tract‐based determinants, those living in a tract with ≥20% vs <5% of persons living below poverty had an AOR of 1.31 (95% CI 1.16‐1.47), those living in a tract with ≥5% vs <5% of persons living in crowded conditions had an AOR of 1.17 (95% CI 1.11‐1.23), and those living in a tract with ≥40% vs <5% female heads of household had an AOR of 1.32 (95% CI 1.25‐1.40).

**Conclusion:**

Census tract‐based determinants account for 11% of the variability in influenza hospitalization.

## 1 INTRODUCTION

Influenza epidemics result in substantial morbidity and mortality each year in the United States with annual deaths ranging from approximately 3300‐49 000.^1^, ^2^, ^3^, ^4^, ^5^ Although annual vaccination is fundamental in preventing and controlling influenza,^6^ vaccination coverage is suboptimal and is further impeded by racial and ethnic disparities.^7^, ^8^ To guide vaccination programs and reduce disease burden, it is essential to understand the risk factors for severe influenza outcomes.

Interventions and policies that focus on both individuals and their neighborhoods may be more effective in improving health outcomes than those that focus exclusively only on individuals.^9^, ^10^ As most disease surveillance systems do not capture sufficient individual socioeconomic characteristics, using census tract data can provide important neighborhood‐level dynamics that impact an individual's risk for influenza to characterize neighborhood‐level determinants.^11^, ^12^, ^13^, ^14^, ^15^, ^16^, ^17^, ^18^ Census tract‐based determinants such as percent living below poverty, household crowding, and female head of household have been associated with influenza hospitalization.^19^, ^20^, ^21^, ^22^

To date, researchers have incorporated census tract‐based determinants into evaluations of univariate associations with influenza hospitalization, which do not account for whether census tract‐based determinants have an independent effect beyond the effect of individual‐level factors. In community‐level data, population characteristics of individuals living within a census tract tend to be correlated, and hence, the assumption of independence among observations is violated. Multilevel analyses take into account hierarchical data structures^23^, ^24^ and enable us to account for this lack of independence, detect multivariate associations, incorporate covariates at both the individual and the geographic level, and model interactions between variables. These models have been used to evaluate health disparities and to describe the relationship between geographic exposures and a wide variety of health outcomes such as diabetes, immunization, obesity, and cancer mortality.^25^, ^26^, ^27^, ^28^, ^29^, ^30^, ^31^

We conducted a multilevel analysis to identify individual and census tract‐based determinants associated with influenza hospitalization using population‐based surveillance data collected from 14 sites across the United States. We hypothesized that census tract‐based determinants such as poverty and household crowding would contribute significantly to the risk of influenza hospitalization above and beyond individual‐level determinants.

## 2 METHODS

### 2.1 Data sources

We used Influenza Hospitalization Surveillance Network (FluSurv‐NET)^32^ data collected from the 2009‐2010 through the 2013‐14 seasons. FluSurv‐NET is composed of the Centers for Disease Control and Prevention's (CDC) Emerging Infections Program, which conducts population‐based surveillance in select counties in California, Colorado, Connecticut, Georgia, Maryland, Minnesota, New Mexico, New York, Oregon, and Tennessee and the Influenza Hospitalization Surveillance Project (IHSP) which conducts population‐based surveillance in select counties in Michigan, Ohio, and Utah. Rhode Island participated as an IHSP site from 2010‐2013. All sites contributed 5 years’ worth of data from 2009‐2013 except for Michigan (2010‐2013), Ohio (2010‐2013), and Utah (2010‐2013) (4 years) and Rhode Island (2010‐2012) (3 years). This network performs surveillance in over 70 counties covering approximately 27 million people (about 9% of the U.S. population). Patients with laboratory‐confirmed influenza‐associated hospitalizations were identified through hospital laboratory and admission databases, infection control logs, hospital discharge data, and weekly calls to catchment area hospitals. For patients with a positive influenza test, medical records were reviewed using a standardized case report form to collect information on demographic characteristics, medical history (including underlying medical conditions), influenza vaccination, clinical course of illness during hospital stay, and treatment with influenza antiviral medications. Data on underlying medical conditions were collected including asthma, chronic pulmonary disease, metabolic disease, cardiovascular disease (excluding hypertension), blood disorders/hemoglobinopathies, neurologic/neuromuscular disease, renal disease, and liver disease. A patient was considered vaccinated if receipt of influenza vaccine occurred at least 14 days prior to hospitalization. Multiple sources, including the medical record, state vaccination registry, the patient's primary care provider, and interview of the patient or proxy, were used to obtain vaccination history.

Data collection was determined by the CDC to be for routine public health surveillance purposes, and thus was not subject to institutional review board (IRB) approval for human research protections. Participating sites submitted the study to their state and local IRBs for review as required.

### 2.2 Study population

In this analysis, we included children and adults with laboratory‐confirmed influenza‐associated hospitalizations who resided in the surveillance areas and were hospitalized <14 days after the positive test during the 2009‐2010 through 2013‐2014 influenza seasons (October 1 – April 30). Hospitalization was defined as an admission to an inpatient ward of the hospital or patients who were kept in observation for more than 24 hours. Laboratory testing for influenza was performed at the discretion of the clinicians providing medical care, and confirmation included any positive result from diagnostic tests available for influenza including reverse transcriptase polymerase chain reaction (RT‐PCR), rapid antigen test, direct or indirect fluorescent antibody staining, viral culture, or serology.

### 2.3 Geocoding and census tract determinants

Each patient's address was geocoded to a latitude and longitude point and linked to a census tract using geocoding software such as ArcMap.^33^ Each site participating in FluSurv‐NET was responsible for geocoding its own data. Census tracts from geocoded data were then merged to the US Census Bureau's American Community Survey (ACS)^34^, ^35^ and aggregated over 5 years (2009‐2013) to obtain census tract‐based determinants. The census tract‐based determinants extracted included information regarding total tract population, race, ethnicity, age, median income, percent who commute, overall tract population density and population density of children less than 5 years of age, and percent living below poverty, percent college‐educated, percent employed, percent with a female head of household, percent of single‐parent households, median income, percent household crowding (defined as numbers of persons per room), percent insured—both private and public. Details regarding how these variables were defined and correspond to the ACS variables are provided in the eAppendix (Table S1).

### 2.4 Outcome and covariates

Our primary outcome variable was hospitalization due to laboratory‐confirmed influenza during the study period. As our goal was to estimate the association of neighborhood and individual‐level characteristics on influenza hospitalization rates, we included covariates at both the individual level and the census tract level. Individual‐level covariates included age (<5, 5‐17, 18‐49, 50‐64, >65 years), race (White, African American, other), gender, and ethnicity (Hispanic or non‐Hispanic). Census tract‐based determinants for the model were chosen *a priori* based on existing literature, biological plausibility, and whether these factors were found to be associated with influenza hospitalization in a prior study using Tennessee data.^19^ When two variables were found to be highly correlated (Spearman's correlation coefficient >0.70), only one was included to avoid inflation of variances. Spearman correlation values between census tract‐based determinants are presented in the eAppendix (Table S2). Census tract‐based determinants used in the analysis were percent below poverty (0‐4.9, 5.0‐9.9, 10.0‐19.9, 20.0+), percent female head of household (0‐19.9, 20.0‐39.9, 40.0+), percent crowding (0‐4.9, 5.0+), population density (categorized into tertiles), percent insured (0‐79.9, 80.0+), and percent college‐educated (0‐39.9, 40.0+). These categorizations were adopted based on previously published standards by the Harvard Public Health Disparities Geocoding Project.^17^

### 2.5 Exclusions

For our analysis, we excluded those without a hospitalization date and those with a missing laboratory diagnostic test for influenza. For the purposes of geocoding, we excluded addresses that could not be geocoded to rooftop accuracy. Additionally, due to new surveillance areas being incorporated over time, for consistency purposes we limited our sample to only those census tracts that remained common through our study period of interest.

### 2.6 Data analyses

We calculated descriptive statistics by site and influenza season. Categorical variables were summarized by frequency and percentage whereas median and interquartile ranges (IQR) were used to describe continuous variables. We computed crude site‐specific annual incidence rates as well as age‐standardized incidence rates by individual factors as well as census tract‐based determinants. The age‐standardized rates were calculated using the 2000 U.S. Standard Population. Health disparity measures such as risk ratio (RR), risk difference (RD), relative index of inequality (RII), and the Concentration Curve for individual factors age, race, gender, and ethnicity and census tract‐based determinant were calculated by site and are presented in the eAppendix (Table S3‐S8).^36^ These methods and interpretation have been previously published by Tennessee.^19^

The dataset used for the analysis of associations had a three‐level cell structure in which individuals were nested in census tracts, which were further nested within sites. A cell is defined as a unique combination of age, race, gender, ethnicity, season, census tract, and site for the population. For every cell, we computed the hospitalization proportion (defined as the number of influenza hospitalizations divided by the population denominator of the catchment area within each site). Cells without any denominator data or zero people residing were not included. Our three‐level hierarchical structure consisted of a total of 1 458 440 cells nested within 5955 tracts that were further nested within the 14 sites.

A logistic regression model using the denominators as weights was used to model this proportion as the outcome. Due to the correlation present between individuals from the same census tract/site, a random‐effect multilevel logistic regression model was used to evaluate the association between the individual and census tract‐based determinants on influenza hospitalization. First, a multilevel unconditional means model was fit using the proportion as the outcome to compute the intraclass correlation coefficient associated with both tract and site. This helped us quantify the proportion of variability associated with tract and site and to determine whether both needed to be included as a random effect in the model. The season variable was also included as a covariate. All statistics were calculated using R version 3.2.4 (R Development Core Team).

To address whether the association between percent poverty and influenza hospitalization was modified based on population density, we included an interaction of the two terms in the model. In addition, we evaluated whether using population density of children less than 5 years old would yield identical results to the overall population density. Finally, to explore the impact of individual‐level covariates such as the presence of asthma, underlying medical conditions, and influenza vaccination status, we refit the model separately on subsets of patients that were indicated to be affirmative for that variable. The model was also fit on the subset of hospitalized patients who did not get vaccinated for influenza.

## 3 RESULTS

### 3.1 Descriptive statistics of influenza‐positive patients

**Figure 1:**
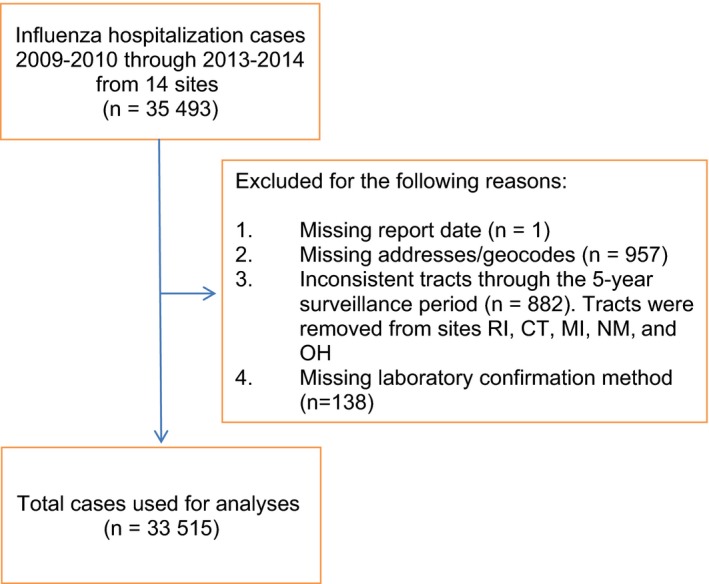
Flowchart of inclusions and exclusions of influenza hospitalization cases 2009‐2010 through 2013‐2014 from 14 sites

Exclusion details are provided via a flowchart in Figure 1 which culminates in 33 515 patients with influenza‐related hospitalization eligible for analysis. The individual‐level and census tract‐based characteristics are shown in Tables 1 and 2, respectively. The 33 515 cases resided in 5713 census tracts (out of a possible 6029 tracts) from 14 sites over the 5‐year study period. The median age of the population was 54 years old (IQR, 28‐72). There were 15 670 (47%) males, 19 495 (58%) were White, 7139 (21%) were African American, and among the 24 429 (73%) that provided ethnicity, 4001 (16%) were Hispanic. The 2012‐2013 season had the highest number of cases (11 224; 33%) and 2011‐2012 had the lowest number of cases (2273; 7%) reported.

**Table 1:** Demographics of 33 515 influenza hospitalization cases 2009‐2010 through 2013‐2014 from 14 sites. Statistics are reported among those that are non‐missing. Categorical variables are summarized by frequency and percentage

Variable	Influenza hospitalizations (%)
aSite	
CA	3517 (10)
CO	3928 (12)
CT	2472 (7)
GA	3358 (10)
MD	3899 (12)
MI	274 (1)
MN	3332 (10)
NM	1444 (4)
NY	3691 (11)
OH	1913 (6)
OR	1990 (6)
RI	853 (3)
TN	1474 (4)
UT	1370 (4)
Age (y)	
<5	3729 (11)
5‐17	2415 (7)
18‐49	8460 (25)
50‐64	7329 (22)
65+	11 582 (35)
Gender	
Male	15 670 (47)
Female	17 845 (53)
Race	
White	19 495 (58)
Black	7139 (21)
Other	6881 (21)
Ethnicity	
Hispanic	4001 (16)
Non‐Hispanic	20 428 (84)
Asthma	
Yes	7512 (22)
No/Unknown	15 820 (47)
Missing	10 183 (30)
+Vaccination status	
Yes	13 043 (39)
No	17 664 (53)
Unknown	2808 (8)
Underlying medical conditions	
Yes	27 814 (83)
No	5157 (15)
Unknown	544 (2)

a All sites contributed 5 y worth of data from 2009‐2013 except for Michigan (2010‐2013), Ohio (2010‐2013), and Utah (2010‐2013) (4 y) and Rhode Island (2010‐2012) (3 y). +Vaccination status stratified by year is provided in the eAppendix (Table S13).

**Table 2:** Census tract‐based determinants of 33 515 influenza hospitalization cases 2009‐2010 through 2013‐2014 from 14 sites. Statistics are reported among those that are non‐missing. Categorical variables are summarized by frequency and percentage

Variable	Influenza hospitalizations (%)
Percent below poverty	
0.0‐4.9	5983 (18)
5.0‐9.9	7373 (22)
10.0‐19.9	9441 (28)
20.0+	10 701 (32)
aPercent crowding	
0.0‐4.9	26 052 (78)
5.0+	7445 (22)
Percent female head of household	
0.0‐19.9	12 316 (37)
20.0‐39.9	11 728 (35)
40.0+	9407 (28)
Population density (persons/mi^2^)	
0‐789	9535 (28)
790‐1959	11 190 (33)
1960+	12 790 (38)
Percent insured	
0.0‐79.9	7377 (22)
80.0+	26 123 (78)
Percent college‐educated	
0.0‐39.9	4981 (15)
40.0+	28 532 (85)

a Percent crowding is defined as having more than one person per room.

### 3.2 Hospitalization rates and Disparity measures

**Figure 2:**
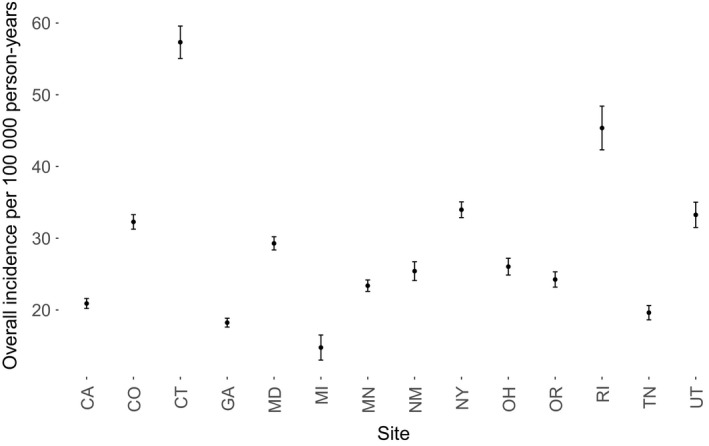
Crude annual influenza hospitalization incidence rate per 100 000 persons by site. The study population includes influenza hospitalization cases 2009‐2010 through 2013‐2014 from 14 sites

**Figure 3:**
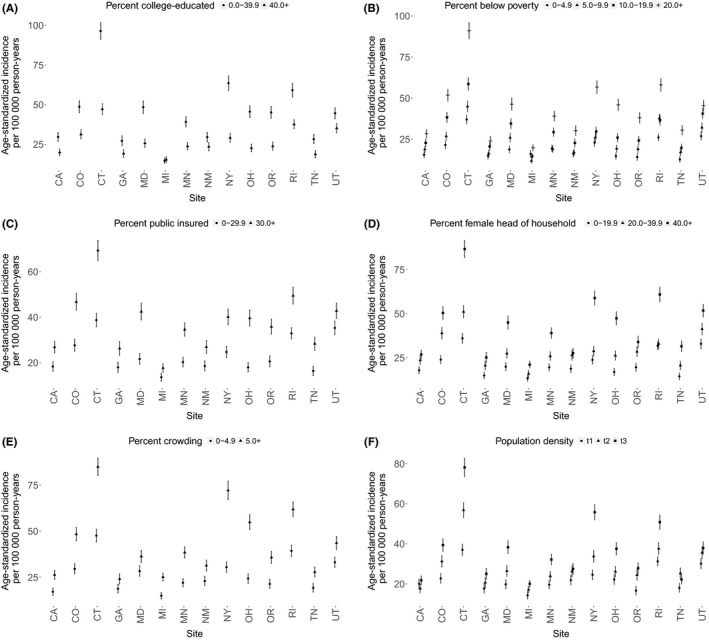
Age‐standardized influenza hospitalization incidence rates by site and census tract‐based determinants (A‐F). The study population includes influenza hospitalization cases 2009‐2010 through 2013‐2014 from 14 sites

Crude annual influenza hospitalization incidence rates per 100 000 persons by site are shown in Figure 2. Age‐standardized rates by site and census tract‐based determinants were computed and are shown in Figure 3(A‐F). Census tracts with greater than 20% of the population living below poverty had the highest rates of influenza hospitalizations across all sites, as did those with greater than 40% of homes having a female head of household. Similar disparities were observed with increases in crowding and population density, and with decreases in percent college‐educated and percent insured.

**Figure 4:**
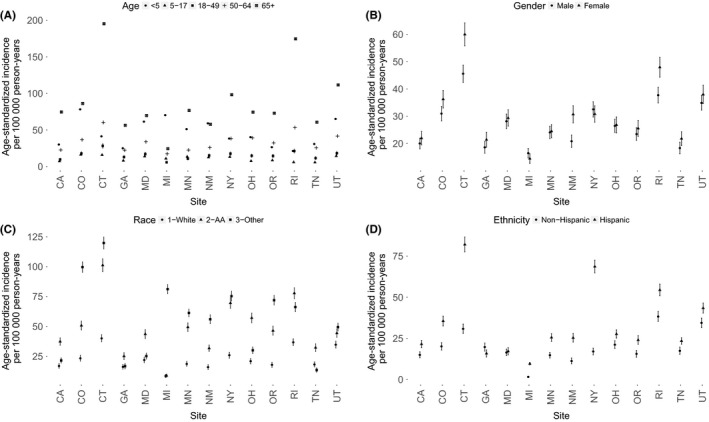
Age‐standardized influenza hospitalization incidence rates by site and individual factors (A‐D). The study population includes influenza hospitalization cases 2009‐2010 through 2013‐2014 from 14 sites

Age‐standardized rates by site and individual‐level factors are presented in Figure 4(A‐D). For individual‐level factors, those 65 years and older had the highest rates of influenza hospitalization. Females and Hispanics also had a higher rate of influenza hospitalization in most sites.

### 3.3 Multilevel model

In the unconditional model that included only the site and tract within site as random effects, the intraclass correlation that describes the cluster effect was computed to be 8% for site and tract and 11% for site alone. This indicated the presence of a moderate clustering effect by site and that 11% of the variability in influenza hospitalization could be explained by census tract‐based determinants. This left 89% of the variability to be accounted for by individual‐level characteristics.

All individual‐level demographics were significantly associated with influenza hospitalization with the highest adjusted odds ratio (AOR) of 9.20 (95% CI 8.72‐9.70) for adults 65 years and older vs children 5‐17 years (Table 3). African Americans had an AOR of 1.67 (95% CI 1.60‐1.73) compared to Whites and Hispanics had an AOR of 1.21 (95% CI 1.16‐1.26) as compared to non‐Hispanics.

**Table 3:** Adjusted odds ratio and its associated 95% confidence interval from the multilevel regression model of influenza hospitalization adjusting for individual‐level (age, gender, race, and ethnicity) and census tract‐based determinants (female head of household, percent below poverty, percent crowding, population density, percent insured, and percent college‐educated)

Model variables	AOR (95% CI)
Age: <5 vs 5‐17	3.99 (3.76, 4.24)
Age: 18‐49 vs 5‐17	1.42 (1.35, 1.50)
Age: 50‐64 vs 5‐17	3.33 (3.15, 3.52)
Age: 65+ vs 5‐17	9.20 (8.72, 9.70)
Gender: Female vs Male	1.14 (1.11, 1.17)
Race: African American vs White	1.67 (1.60, 1.73)
Race: Other vs White	1.31 (1.26, 1.36)
Ethnicity: Hispanic vs Not	1.21 (1.16, 1.26)
Female Head of Household: 20.0‐39.9 vs 0‐19.9	1.14 (1.09, 1.19)
Female Head of Household: 40.0+ vs 0‐19.9	1.32 (1.25, 1.40)
Percent Below Poverty: 5.0‐9.9 vs 0.0‐4.9	1.14 (1.05, 1.24)
Percent Below Poverty: 10.0‐19.9 vs 0.0‐4.9	1.20 (1.10, 1.31)
Percent Below Poverty: 20.0+ vs 0.0‐4.9	1.31 (1.16, 1.47)
aPercent Crowding: 5.0+ vs 0‐4.9	1.17 (1.11, 1.23)
Population Density: Tertile 2 vs Tertile 1	1.06 (0.97, 1.15)
Population Density: Tertile 3 vs Tertile 1	1.07 (0.95, 1.21)
Percent Insured: 0.0‐79.9 vs 80.0+	1.06 (1.01, 1.12)
Percent College‐Educated: 0.0‐39.9 vs 40.0+	1.14 (1.07, 1.20)

a Percent crowding is defined as having more than one person per room.

All neighborhood measures except for population density were significantly associated with influenza hospitalization. Patients living in a census tract with more than 20% vs <5% of persons living below poverty level had an AOR of 1.31 (95% CI 1.16‐1.47). Those living in a census tract with over 40% vs <20% of persons with a female head of household had an AOR of 1.32 (95% CI 1.25‐1.40). Persons living in a tract with more than 5% vs <5% living in crowded conditions had an AOR of 1.17 (95% CI 1.11‐1.23).

### 3.4 Sensitivity analyses

**Figure 5:**
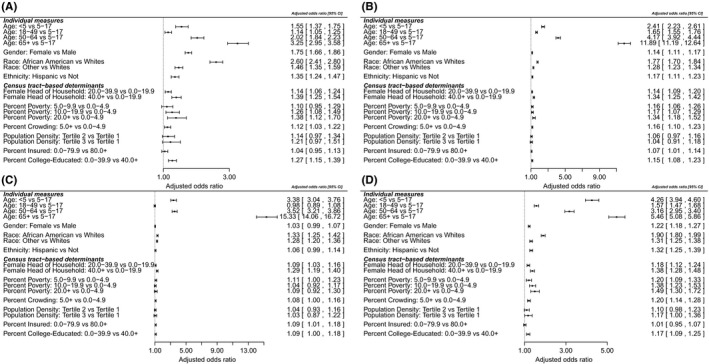
Forest plots displaying adjusted odds ratio and its associated 95% confidence intervals for the subset of cases that had positive indicators for asthma (A), underlying conditions (B), vaccination (C), and no vaccination (D). The study population includes influenza hospitalization cases 2009‐2010 through 2013‐2014 from 14 sites

As part of sensitivity analyses, we evaluated whether the association between percent poverty and influenza hospitalization was modified based on population density by including an interaction of the two terms in the model. We also evaluated whether using population density of children less than 5 years old would result in the same results as including overall population density. The model results remained similar in inference when using population density of children less than 5 years old in place of overall population density. The interaction between population density and percent of people living below poverty was not statistically significant and hence was removed from the model for parsimony and ease of interpretation. We conducted stratified analyses on subsets of the data that included asthma, underlying medical conditions, and influenza vaccination status. The model was also fit on the subset of patients that indicated that they did not get vaccinated for influenza. Among people diagnosed with asthma, those who were not vaccinated for influenza as well as those with underlying conditions, the three separate models provided approximately similar results to the overall population model with percent below poverty being associated with influenza hospitalization. However, in the model with hospitalized persons vaccinated for influenza, the association of influenza hospitalization with percent below poverty was no longer observed. The forest plots of the adjusted odds ratios for these subsets are provided in Figure 5(A‐D).

## 4 DISCUSSION

In this study, we adopted a multilevel modeling approach in order to better understand the simultaneous contributions of individual and census tract‐based determinants on influenza hospitalization while accounting for geographic clustering. In our analyses of 33 515 influenza hospitalizations from 14 sites over a 5‐year period, we found that both individual and census tract‐based determinants were associated with hospitalization with laboratory‐confirmed influenza. The results were consistent with recent studies that demonstrated that living in a neighborhood with a high percentage of residents below the poverty line is a risk factor for influenza hospitalization.^19^, ^20^, ^21^, ^22^ Specifically, in our study the odds of being hospitalized with influenza were 31% higher in census tracts with the highest level of poverty as compared to those tracts with the lowest level of poverty. In addition, neighborhoods with the highest percent of female head of households were 32% more likely to be hospitalized with influenza as compared to those with the lowest percent. Furthermore, living in a census tract with a high level of household crowding was associated with higher odds of influenza hospitalization.

Our unconditional model indicated that 11% of the variability in influenza hospitalizations could be explained by clustering within a census tract leaving 89% of the variability to be accounted for by individual‐level characteristics. Individual‐level characteristics such as age, race, gender, and ethnicity were all associated with influenza hospitalization with the highest association observed in those >65 years old. These findings were also consistent with the univariate analysis across all the sites, suggesting that these results were robust. In the sensitivity analysis, individual models limited to people diagnosed with asthma, those who were not vaccinated for influenza, and those with underlying conditions had approximately similar results for individual and census tract‐based determinants (Figure 5) compared to the overall population model. However, when evaluating those persons who had received influenza vaccination, the association of influenza hospitalization with percent below poverty was no longer present. Vaccination has resulted in improvement in the racial differences seen in invasive pneumococcal disease for example,^37^, ^38^ and hence, this finding warrants further investigation.

The multilevel framework has been used to evaluate determinants of area‐level and individual‐level inequalities in public health to quantify the relative contribution of geographic, demographic, and socioeconomic factors. A major rationale for using the multilevel approach in the evaluation of influenza hospitalization is that interventions and policies that focus on individuals and the neighborhood environment may be more effective in improving public health than interventions that focus on just individuals. It can also prioritize individual interventions (such as vaccinations) to certain high risk census tracts. The multilevel framework allows us to evaluate the association between influenza hospitalization and predictors at both levels. This modeling strategy also enables us to partition the variability attributable to the individual‐level and the census tract determinants allowing us to quantify the contribution of the census tract‐based determinants in the variation in influenza hospitalization rates.

Other notable strengths of this study include the following: (i) geocoding of data which includes indicators that are not routinely captured in surveillance systems; (ii) the use of census tract‐based determinants which detect socioeconomic gradients more consistently than zip codes; and (iii) a large cohort of 33 515 population‐based laboratory‐confirmed influenza hospitalizations over a period of 5 influenza seasons from 14 sites geographically dispersed across the United States.

Limitations to our study should also be noted. Testing practices (low testing rates or use of tests with low sensitivity) could vary by census tract which may bias influenza hospitalization rates and this was not accounted for in our analyses. Our measures of census tract‐based determinants were categorized *a priori* based on existing cutoffs. This resulted in some variables having very few events which may affect the precision of estimates. We also had data regarding hospitalizations, necessitating development of a control group extracted from the census data—which would have possibly contained those with influenza, but did not develop enough complications to be hospitalized. We thus were only able to conduct analysis on the associations of individual covariates such as vaccination status using a subset of patients, and this may lead to biased estimates of association. Due to our control group possibly containing those who had influenza but were not hospitalized, the impact of socioeconomic determinants in our analysis may be underestimated. Although this study uses census tract‐based determinants as a proxy for individual socioeconomic measures, we acknowledge the possibility of ecological fallacy and that these determinants provide information only regarding the neighborhood that are not reducible to the individual level. Additionally, residents from nursing homes and other long‐term care facilities as well as patients transferred from other hospitals were not excluded from the analyses, so the analyses results may not completely represent findings for community‐dwelling adults. Although our results may be relevant to the population of the United States, the results found here may not have generalizability to other countries and care must be taken when attempting to generalize these results to populations outside the United States.

Area‐based socioeconomic determinants have been shown to be strongly associated with a wide range of health problems, and hence, our finding that socioeconomic determinants play a fundamental role in influenza hospitalization is not alarming. Although the exact underlying mechanisms remain unclear, we hypothesize that socioeconomic determinants probably shape individual behavior by influencing characteristics such as individual education, their income, medical insurance status, education, access to care, and health‐related behaviors as well. Factors such as crowding may also increase exposure to the influenza virus. It is likely that there exist possible different perceptions of the seriousness of influenza and hence delays in seeking care that may be related to the outcome as well. It can also be hypothesized that high level of health literacy may be associated with areas with higher percent of educated.

In conclusion, although the strongest associations were observed with respect to individual‐level characteristics such as age and race, census tract‐based determinants also were associated with hospitalization for influenza. Identifying and targeting areas for specific prevention and control interventions (eg, vaccination) could help reduce some disparities in influenza outcomes for areas with high percent of poverty, household crowding, and female heads of households.

## DISCLAIMER

The findings and conclusions in this paper are those of the author(s) and do not necessarily represent the views of the Centers for Disease Control and Prevention.

## CONFLICT OF INTEREST

None declared.
